# A new way of thinking? Exploring professionals’ perspectives on the sustainability of interprofessional team meetings: A focus group study

**DOI:** 10.1080/13814788.2026.2708526

**Published:** 2026-08-10

**Authors:** Jelena Slowig, Darcy Ummels, Emmylou Beekman, Marloes van Bokhoven, Albine Moser

**Affiliations:** ^a^ Research Centre for Autonomy and Participation in Health Care and Social Welfare, Zuyd University of Applied Sciences, Heerlen, The Netherlands; ^b^ Department of Family Medicine, Care and Public Health Research Institute (CAPHRI), Faculty of Health, Medicine & Life Sciences, Maastricht University, Maastricht, The Netherlands; ^c^ Department of Rehabilitation Medicine, Care and Public Health Research Institute (CAPHRI), Faculty of Health, Medicine & Life Sciences, Maastricht University, Maastricht, The Netherlands; ^d^ Living Lab Ageing and Long-Term Care, Care and Public Health Research Institute (CAPHRI), Faculty of Health, Medicine & Life Sciences, Maastricht University, Maastricht, The Netherlands

**Keywords:** Qualitative research, sustainability, interprofessional collaboration, primary care, older adults

## Abstract

**Background:**

Interprofessional collaboration is essential to address the complex challenges in primary care. One widely used method for this purpose is interprofessional team meetings. Although the benefits are generally known, maintaining these meetings after their introduction remains challenging.

**Objective:**

To understand the aspects influencing the sustainability of interprofessional team meetings.

**Methods:**

An exploratory qualitative study was conducted with professionals (*n* = 39) from seven interprofessional teams that met regularly to coordinate elderly patient care. Data were collected through seven focus group discussions. Content analysis was applied, guided by the Consolidated Framework for Sustainability, to identify barriers, facilitators, and needs.

**Results:**

Common barriers included unclear networks, lack of shared goals, and limited time, whereas facilitators involved strong team dynamics, clear structures, and flexibility. These findings point to five key needs: (i) adaptive team composition in response to patient care issues, (ii) shared ownership within interprofessional teams, (iii) shared meetings’ aims and work agreements, (iv) a balanced structure with clear procedures, supportive tools and room for flexibility, (v) and a satisfactory perceived quality of the meetings.

**Conclusion:**

This study shows that nurturing team dynamics, tailoring organisational structures, and embedding ongoing evaluation are essential beyond patient care. This may need a new way of thinking to enhance the sustainability of interprofessional team meetings in primary care.

## KEY MESSAGES

- Reflection within interprofessional teams helps to understand aspects that influence the sustainability of meetings.
- Creating a safe space for open communication and negotiating goals and expectations may enhance sustainability.
- Interprofessional meetings should be embedded in the organisational structure with clear tasks, procedures, responsibilities, and sufficient time should be allocated for participation.

## Introduction

Interprofessional (IP) collaboration is essential to address the current and future challenges of primary care, including the complex health conditions of the ageing population and the simultaneous shortage of healthcare professionals (HCPs) [1]. In response to these challenges, Dutch and European policies promote independent living at home for as long as possible, both to match older adults’ preferences and to reduce pressure on inpatient care. This has become central to people-centered, integrated, and appropriate healthcare models [2]. As a result, continuous care coordination of patients’ health is required involving teams of HCPs with different backgrounds engaging in IP collaboration [3]. These IP teams typically include general practitioners (GPs), nurses (such as practice nurses, district nurses, and advanced nurse practitioners-ANPs), pharmacists, social workers, and allied HCPs, such as occupational or physical therapists. The World Health Organisation defines IP collaboration as health workers from different backgrounds working with patients, families, and communities to provide high-quality care [4]. Building on this, Morgan and colleagues introduced IP teamwork, a more integrated form of collaboration based on interdependence [5]. This study focuses on IP teamwork, especially on regular IP team meetings that aim to coordinate IP care goals, plans, and interventions [6,7]. In the Netherlands, the IP team meeting is a typical form of IP teamwork, usually organised and held within GP-practices.

Empirical findings show positive outcomes of IP teamwork at three levels for: patients (e.g., satisfaction, adherence), HCPs (e.g., job satisfaction, reduced burnout), and organisations (e.g., trust, shared goals) [8,9]. Despite its proven benefits, sustainability of IP teamwork remains challenging, as numerous studies show that it often faces discontinuation [10–12]. In this study, sustainability is defined as the maintenance of IP team meetings, including their outcomes, as well as the processes involved in adapting IP team meetings in response to emerging needs in the context [13,14]. A gap between initial implementation and long-term sustainability of healthcare initiatives has been highlighted [15]. The challenge lies in bridging this gap, ensuring that IP team meetings remain sustained over time after initial implementation. Although systematic reviews describe strategies to support implementation of IP collaboration, they do not specifically address strategies for regular IP team meetings [8,16]. These studies also highlight a lack of knowledge about whether these strategies contribute to long-term sustainability and emphasise that strategies cannot be generic but must be tailored to specific contexts [16]. This raises the research question: What are the perceived needs, facilitators and barriers of primary HCPs to foster sustainability of their IP team meetings? The aim is to understand the complexity of aspects influencing the sustainability of IP team meetings, as a prerequisite for developing impactful context-specific sustainability strategies.

## Methods

### Research design

We conducted an exploratory qualitative study, collecting data by focus group discussions (FGD) [17]. Theoretical orientation was provided by the Consolidated Framework for Sustainability (CFS) [13]. The CFS was specifically developed to capture key constructs underlying sustainability in healthcare. It synthesises 62 existing approaches, including widely used models such as the Practical, Robust Implementation and Sustainability Model (PRISM) and the Dynamic Sustainability Framework, resulting in a comprehensive framework comprising six main constructs: initiative design and delivery, negotiating processes, people involved, resources, organisational setting, and external environment.

### Study context

This study took place in a primary care health trust in the south of the Netherlands, covering both rural and (sub-)urban settings. A total of 114 GPs are supported through this trust, called HOZL (General Practitioners Eastern South-Limburg). The population is one of the most ageing regions in the Netherlands, characterised by a high prevalence of chronic conditions and a lower socio-economic status.

### Sample & recruitment

The participants were HCPs from various backgrounds. Inclusion criteria were being part of an IP team meeting that met regularly to coordinate care for vulnerable older adults and included HCPs from at least three professions (e.g., GPs, elderly care physician, nurses, therapists).

A two-step sampling approach was applied. First, convenience sampling was used within the HOZL region to select eligible IP teams. Study information was disseminated *via* the HOZL newsletter, Microsoft Teams channel, and direct communication during network meetings. Second, total population sampling was applied within the included IP teams: all HCPs present were eligible and invited to participate in a FGD. Invitations were sent both in writing and personally by the research team.

### Data collection

We collected data from April 2023 to May 2024. We developed the FGD questioning route based on five of the six CFS-constructs [13,17]. The construct initiative design and delivery was not applied, as the study focused on perceived needs, facilitators, and barriers rather than on the design, implementation, or effectiveness of specific strategies. Open-ended questions addressed facilitators, barriers, and needs, with probes used to explore the relevant CFS constructs (see Box 1; Appendix 1). Facilitators and barriers represent existing aspects that, respectively, support and hinder sustainability, whereas needs reflect aspects that could foster sustainability in the future.

Box 1. Main questions focus group discussion

**Table:** 

Facilitators	What keeps your interprofessional team meeting going? What should continue to happen as it does now?
Barriers	Which barriers need to be addressed in order to ensure the sustainability of your interprofessional team meeting?
Needs	What do you need for sustaining your interprofessional team meeting? What is currently missing for the sustainability of your interprofessional team meeting?

The FGDs were held with each IP team by two researchers (JS and DU) in the GP-practices. They lasted, on average, 50 min. One researcher (JS) acted as the moderator, while the second researcher (DU) took field notes. The moderator facilitated the discussion by encouraging active and balanced participation among all participants, explicitly inviting contributions from less vocal participants when needed. The co-moderator observed non-verbal behaviour, including signs of hesitation or disagreement, and used these observations to seek clarification during the discussion. The FGDs were audio-taped and transcribed verbatim.

### Data analysis

We conducted a systematic deductive content analysis as outlined by Elo and Kyngäs [18]. First, the transcripts were read several times to become thoroughly familiar with the data. We then developed an unconstrained categorisation matrix based on the predefined constructs of the CFS [13] (Appendix 2). The categories of the CFS were used as an initial coding framework, while remaining open to data-driven refinement during analysis to ensure that participants’ experiences were accurately and richly represented. The first FGD was independently coded by two researchers (JS, DU). One researcher (JS) analysed the remaining FGDs, after which the analyses were reviewed, discussed, and refined by the whole research team. Finally, the findings were synthesised within five of the six CFS constructs: negotiating initiative processes, people involved, resources, organisational setting, and external Environment. These were converted into building blocks that may enable other IP teams to reflect on the sustainability of their own meetings. We used analysis software (ATLAS.ti 25) to organise data.

### Ethical considerations

All participants provided verbal informed consent before taking part. Confidentiality was ensured by pseudonymizing all data, removing identifying information, and reporting findings in a way that prevents identification of participants or teams. Data were stored securely and accessible only to the research team. Ethics approval for our study was received from the Medical Ethical Review Committee of Zuyderland, the Netherlands (METCZ20220095).

### Trustworthiness

To ensure trustworthiness, we applied several strategies [19]. We enhanced credibility through member checking, source triangulation (transcripts, reflexive notes, and field notes), and analyst triangulation supported by repeated peer debriefings and reflection sessions within the research team, during which both the adequacy of the categorisation matrix in capturing the richness of the data and the attainment of data sufficiency were iteratively discussed. We provide detailed contextual and participant descriptions to support readers in assessing the transferability of our study process and findings to their own settings.

## Results

Tables 1 and 2 show the characteristics of the 39 participants included in this study and the seven IP team meetings. Team composition varied between meetings, with nurses participating in all seven meetings, GPs in six, allied HCPs in three, and elderly care physicians in two. Shared agendas were used in 3 IP team meetings, and 6 meetings were chaired to structure content and time. None of the meetings included shared reporting after meetings. Patients did not participate in IP team meetings, as professionals indicated that conversations with patients and their relatives typically take place afterwards during home-based consultations, in which the relevant professionals are involved to discuss care options and support shared decision-making.

**Table 1.** Characteristics of participants (*N* = 39).

Characteristics	Number (%) of professionals, unless other specified
**Gender**	
Female	30.0 (76.9%)
Male	9.0 (23.1%)
Mean age in years* (sd; range)	44.1 (12.0) (23.0–70.0)
Mean work experience in years* (sd; range)	16.3 (11.2) (1.0–44.0)
Education*	
University	10 (26.3%)
University of Applied Sciences	23 (60.5%)
Vocational education	5 (13.2%)
**Professionals’ disciplines and specialties**	
**Medical:**	
General Practitioner	8 (20.5%)
Elderly care physician	2 (5.2%)
**Nurses:**	
GPs Practice nurse	7 (17.9%)
Advanced nurse practitioner (ANP)	2 (5.2%)
District nurse	11 (28%)
Case manager dementia	6 (15.4%)
Palliative nurse	1 (2.7%)
**Allied HCPs:**	
Physical therapists	2 (5.1%)

*1 missing value.

**Table 2.** Characteristics of interprofessional team meetings (*N* = 7).

Characteristics	Number of meetings, unless other specified
**Professionals present during meetings**	
Medical professionals	
General practitioner	6
Elderly care physician	2
Nurses	7
Allied health care professionals	3
**Setting of team meeting**	
Urban	3
Suburban	3
Rural	1
**Mean meeting duration in minutes (range)**	66 (60–90)
**Mean meeting frequency in weeks (range)**	7 (1–12)

In total, seven FGDs were conducted, one with each included IP team. Table 3 presents the needs, facilitators and barriers corresponding to five CFS constructs [13]. Most identified needs were consistently reported across all FGDs.

**Table 3.** Barriers, facilitators, and needs identified within five main constructs of the consolidated framework for sustainability (CFS) [13].

CFS	Identified barriers, facilitators and needs	Building Blocks
**People involved**	**Barriers:** Uncertainty about interprofessional team composition; Unfamiliarity with colleagues in the networks **Facilitators:** Positive team dynamics (e.g., trust, open communication, collective decision making) **Needs:** Presence of relevant professionals (e.g., case and problem specific invitation of professionals and personally getting to know colleagues and their expertise); Shared ownership/autonomies leadership (e.g., clarity about shared responsibilities and ownership, key figures, chairpersons); Patient communication roles (agreements about responsibilities around communication with patients before and after meetings)	A team culture characterised by safety, open communication, and shared decision-making. Adaptive team composition: Flexible invitation of relevant professionals based on patient care needs. Shared ownership among team members for both the process and outcomes of interprofessional team meetings.
**Negotiating processes** **&** **Organisational setting**	**Barriers:** Lack of shared aims and agreements (e.g., disagreements within interprofessional teams); Lack of perceived quality and usefulness (e.g., unclear content to discuss during meetings); Knowledge gaps (e.g., uncertainties about how to adapt team meetings and respond to challenges); non-delegable tasks (e.g., accessing information in the general practioners’ patient-file) **Needs:** Negotiating processes on shared aims and expectations regard all aspects of interprofessional team meetings (e.g., team compositions, expectations, responsibilities, meetings’ procedures); Knowledge and best-practice examples (e.g., to support the development of sustainable solutions for team meetings); Meetings’ evaluations	Negotiating: Collectively and clearly defined goals, expectations, and work agreements to ensure alignment and continuity. Ongoing, structured evaluation that enables early identification of sustainability challenges, thereby enhancing the perceived quality and value of interprofessional team meetings.
	**Barriers:** Excessive formalisation (e.g., strict procedures and formats, time intensive demands before meetings) **Facilitators:** Flexibility (e.g., adaptations to emerging needs); shared work agreements & tools (e.g., Clear documentation of decisions, annual meetings schedules, meetings agendas incl. clearly defined problems to solve); Handover structures (e.g., information-sheets/folders for potential new team members outlining routine processes and work agreements) **Need:** An efficient yet balanced structure between clear procedures, supportive tools and flexibility based on the negotiated preferences of the interprofessional team.	Organisational structures (such as procedures, tools, and tasks) that are flexible enough to accommodate ad hoc changes.
**Resources**	**Barriers:** Insufficient time to focus on topics beyond direct patient care (e.g., interprofessional collaboration quality and meetings’ processes, to actively involve patients, to prepare content for meetings beforehand, to evaluate meetings); Funding (e.g., lack of funding limits attendance during meetings); Staff (e.g., limited availability of professionals due to staff turnover, part-timers, and an overall shortage of professionals); Infrastructures (e.g., lack of a unified communication system, shortage of sufficiently large meeting rooms) **Needs:** Additional administrative staff (e.g., a dedicated secretary to manage the logistical and administrative aspects of the meetings); Infrastructures (e.g., suitable meeting space; shared digital communication system accessible for whole interprofessional team.	Availability of time, financial resources, appropriate meeting space, digital communication infrastructure, and administrative staff.
**External environment**	**Barriers:** Lack of a protocol for complying with privacy regulations **Needs:** Guidelines and practically feasible approaches for complying with privacy regulations	Compliance with the General Data Protection Regulation to ensure the secure handling of patient data in connection with interprofessional team meetings.

### People involved: Interprofessional team

Multiple HCPs reported uncertainty regarding which disciplines should participate in IP team meetings, and whether on a permanent or flexible basis. Participants reported that the presence of HCPs relevant to addressing a patient’s specific care issues is essential. They indicated that elderly care physicians, allied HCPs, or palliative care nurses are often missing from the meetings. The networks linked to GP-practices are perceived as large, owing to the diversity of therapy and outpatient care services, particularly in (sub-)urban regions, making it challenging for GPs to invite all relevant HCPs for a given patient case. Consequently, relevant HCPs attend irregularly, and IP team compositions are perceived as fragmented. Participants expressed the need to personally get to know stakeholders to gain a clearer overview of the network, as well as case-specific invitations for relevant HCPs who do not attend regularly.

IP team 7 ‘Linked to one general practice, you have […] lots of district nurses, different physical therapists, so of course it is difficult to get all that insight for each patient.’

While ANPs and practice nurses, employed at the GP-practice, are often perceived as key figures for ensuring continuity of the IP team meetings, their central role also reveals a vulnerability: without them, meetings frequently do not take place. This highlights the need for shared ownership within IP teams, so that the meetings can continue even in the absence of them.

IP team 4 ‘When you [ANP] are on vacation, we do not have meetings either. […] Suppose you [ANP] cannot attend, then we should still sit here together. […] So, I think it [the meeting] becomes self-directed. And that is also what you need to keep the meetings going.’

Participants described team dynamic related facilitators for sustaining IP team meetings, including mutual trust, personal contact, collective decision-making, and open communication. A reciprocal facilitating dynamic is described: Familiarity through in-person contact fosters commitment to and collaboration within and beyond IP team meetings, while unfamiliarity is seen as a barrier to their sustainability.

IP team 1 ‘But you also find each other faster because we have these meetings, right? I think that is why we have such short lines of communication.’

### Negotiating processes & organisational setting

The themes within these constructs showed substantial overlap. As a result, the findings are presented in a synthesised manner.

#### Team meetings’ common aims and work agreements

A lack of shared aims among HCPs is a recurring barrier across several IP teams. Repeated disagreements within IP teams during the FGDs indicated that members’ expectations and aims were not always aligned.

IP team 5: GP1; ‘Imagine the patient joins our meeting and does not know anyone at the table, which is probably quite uncomfortable for them. The idea that everyone is talking about you, right?’ GP2; ‘But do you think the patient would actually feel comfortable with people talking about them behind their back?’

Most IP teams reported that they had never negotiated on shared aims or established work agreements. In many cases, the team members responded with shy laughter or appeared unsure of how to approach the topic during the FGD. The need emerged for a negotiating process on shared aims and agreements for all aspects of the IP team meeting.

IP team 6: Moderator; ‘How clear are your shared aims to each other? Have you documented them or discussed them explicitly?’ GP1; ‘No, that has been tacitly assumed [group laughs softly]. No, we have not, no of course not [firmly voiced], nothing has been put down on paper.’

Low perceived quality of IP team meetings acts as a barrier of sustainability, often due to urgent issues being handled outside meetings, discussing unknown patients, repetitive cases without progress, or passive attendance of individual HCPs.

IP team 6 ‘At one point, we also started having a biweekly meeting with home care, because we were getting a lot of the same questions every day. But then we started wondering what is the purpose of the IP team meeting? It felt like the effectiveness was waning and that was pretty confusing.’

IP teams that described their meetings as lacking structure and overly informal reported doubts regarding their overall quality. These teams expressed uncertainties about how to respond to challenges and voiced, implicitly and explicitly the need for knowledge and best-practice examples to support the development of sustainability solutions for their IP team meetings.

#### Team meetings’ procedures and adaptability

An interrelation between well-structured and ad-hoc organisation of IP team meetings is recognised within and across the 7 IP teams. This is sometimes experienced as a barrier, and other times as a facilitator. Participants emphasised that, while too much formalisation can be disengaging, having clear procedures, such as shared meetings’ agendas and explicit decisions about who to invite for specific cases, supports the sustainability of IP team meetings. In the absence of such procedures, HCPs experienced task and role confusion. At the other hand some IP teams appreciated ad-hoc agenda setting to reduce preparation time. Flexibility was identified as a facilitator across several IP teams. Teams appreciated the ability to adjust the meeting agenda for urgent cases, to cancel meetings when no patient cases required discussion, or passing information *via* colleagues when someone is absent.

IP team 5 ‘And then we just see, if someone says, ‘Hey, I only have one case. Can I start with that?’ we take a look and decide. In that sense, we are flexible. We check whether there are external professionals who also want to present their case, and then adjust accordingly. You know, I think that is the kind of flexibility you need.’

Across all IP teams, participants expressed the need for a balanced structure that combines clear procedures and task allocation with sufficient flexibility and informality. Many HCPs were convinced that IP team meetings first require adequate organisational embedding through clear procedures and streamlined processes in order to be sustained.

IP team 6 ‘An IP team meeting needs to be efficient and well-documented. Yes, the goals must be clear, and it should be formalised and professionalised. […] I usually say ‘Let us focus on delivering good care first, and the rest will follow’. But when it comes to the IP team meeting, if you add up all the hours we spend on it together, it simply has to be done well and as streamlined as possible’.

#### Supportive tools

Several tools support the sustainability of IP team meetings, such as annual schedules, agendas, information folders for new chairs, minutes, and clear backup contacts in case of absence. Participants emphasised the need for templates for these tools. Additionally, several IP teams voiced the need of conducting structural evaluations of meetings to guide ongoing adjustments, as well as establishing participation agreements and handover folders.

IP team 2 ‘What I have done is create a folder with information about the meeting routine, which includes sending out the agenda two weeks in advance. We have fixed agenda items […] Everything is documented in this folder, and a new colleague will need to check if she can manage it this way’.

### Resources

HCPs lack time to address topics beyond immediate patient care, such as working on sustainability or to actively involve patients. Preparing meeting content and attendance during meetings often does not fit into schedules. Participants stressed the need of starting and ending meetings punctually and for additional time to adequately organise the meetings.

IP team 4 ‘Yes, I have written down some meetings’ work agreements and tasks of our meeting, but they were never shared. We have never really, well, discussed this in detail. And honestly, we do not have time for that either’

Staff-related aspects hinder the sustainability of IP team meetings such as part-time work or shortage of HCPs. All IP teams report that staff turnover disrupts continuity. HCPs emphasised the need for a dedicated secretary to manage the administrative aspects of the meetings.

IP team 6 ‘Speaking of continuity, it is just a problem […] the care is overstretched and under pressure from all sides, I am not surprised that some colleagues have dropped out. But it does mean we are missing a big part of the continuity in our team again’.

Most HCPs cited the lack of a digital shared patient system as a major barrier, in which all disciplines involved in the patient’s care can jointly access each other’s reports. Since a shared digital system was perceived as a facilitator for sustainability, some IP teams described it as a need.

IP team 1 ‘Because if it is a colleague from within our organisation, they also report in our system, and we can take it to the meeting. We have the information then, […] so they do not have to join the meeting themselves’.

### The external environment

HCPs voiced the need for guidance on how to comply with privacy regulations in relation to IP team meetings. In the past, the imposed regulations have even led to the elimination of an IP team meeting.

IP team 3 ‘Back then, we were a large group. But then privacy law came, and we were not allowed to discuss every patient with everyone at the table […], so we scrapped the whole meeting’.

## Discussion

### Main findings

This study aimed to understand the complexity of aspects influencing the sustainability of IP team meetings. The most frequently reported barriers included unclear networks and unfamiliarity among team members, excessive formalisation, lack of shared goals, and insufficient time to prepare meeting content and to address topics beyond direct patient care. In contrast, key facilitators were positive team dynamics characterised by trust and collective decision-making, stable team compositions, flexible application of meeting structures, clear procedures and task division, and high perceived meeting quality. Based on the perceived barriers and facilitators, five key needs emerge from our findings: (i) adaptive IP team composition in response to patient care issues, (ii) shared ownership within IP team, (iii) shared meetings’ aims and work agreements, (iv) a balanced structure with clear procedures, supportive tools and room for flexibility, (v) and a satisfactory perceived quality of the meetings.

### Comparison with existing literature

Our findings align with previous studies, which emphasise that building primary care teams is challenging due to the varied problems of older adults, multiple professional disciplines, and diverse institutions [20,21]. As a result, IP team composition requires continuous adaptation rather than stability [20–22]. Coordinating this process is often unfeasible within existing workloads, underscoring the need for external support [23,24].

Furthermore, HCPs expressed the need for shared ownership. This aligns with previous research highlighting that leadership is essential for guiding the IP team meeting process and maintaining the network [25]. Shared ownership may function in IP team meetings as a form of informal leadership, as reflected in a framework comprising four roles: initiator, visionary, tutor, and overseer [26].

**Figure 1.**
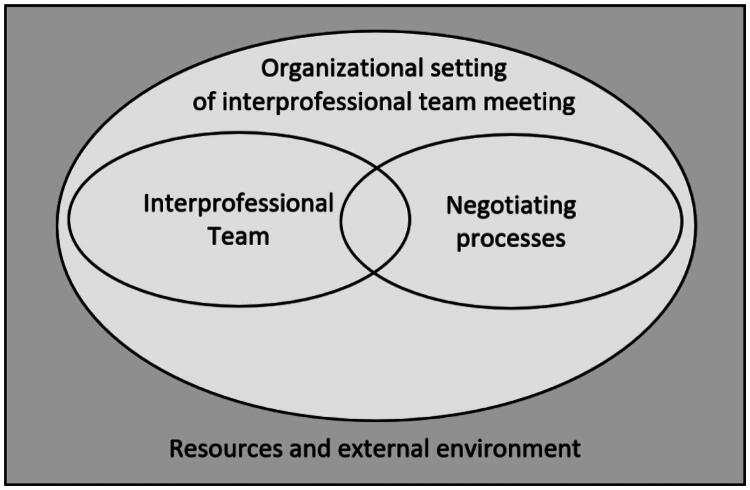
Interrelations of the consolidated sustainability framework constructs in interprofessional team meetings: The outer level (dark grey) represents aspects outside the scope of influence of the interprofessional team, yet strongly influencing the sustainability of the meetings. The organisational setting of the meetings (light grey) is perceived by professionals as a prerequisite for sustainability and lies within the team’s scope of influence. This layer surrounds the interprofessional team and can be shaped through the team’s negotiation processes and interactions.

Our findings suggest that a balance between structured procedures and flexibility in IP team meetings forms an overarching prerequisite for sustainability. HCPs emphasised that a lack of structure undermines meeting quality, while prior studies similarly highlight the interplay between organisation and situational flexibility [25,27]. Some studies go further, concluding that standardising procedures, including their organisational embedding, appears essential for sustainability. However, although standardisation seems crucial, teams often perceive little need for it, and alignment among members is required before any standardisation can occur [10,21]. Participants reported limited experience with negotiating issues beyond direct patient care, suggesting a need for a ‘new way of thinking’ within IP teams. This would create space for negotiation and reflection, enabling teams to develop context-sensitive, balanced structures aligned with the key sustainability needs identified in our study [12,28]. Notably, within this space for negotiation, teams may need to consider how patient perspectives are incorporated, particularly given that participants themselves expressed differing views on whether direct patient participation in IP team meetings is appropriate. While direct patient involvement was not identified as a need for sustainability, it seems closely linked to perceived meeting quality, as participants emphasised the importance of communication with patients before and after meetings, ensuring that patient care needs are adequately represented in meeting discussions (Table 3). However, more research is needed to understand how IP teams can implement the ‘new way of thinking’ in their practice. The aspects mentioned above appear to be partly interrelated and to fall within the team’s scope of influence (Figure 1).

Regarding our findings on the external environment, prior research emphasises that a team-friendly healthcare system is essential for sustainability [29]. This requires structural changes, including appropriate funding models and support from health insurers, municipal organisations, as well as clear policy agreements and long-term strategies that support IP teamwork, such as IP team meetings [25,29].

While this study focused on sustainability, the distinction between implementation and sustainability was not always clear. Some of the identified aspects, such as the need for shared ownership or clear agreements, are equally relevant during the initial implementation [8]. As such, it remains challenging to determine whether these aspects support initial implementation, sustainability, or both. It is conceivable that these aspects were never fully established during the initial implementation, or that they diminished over time due to the barriers described [15]. As an IP team meeting, it is worth considering whether the agreements established during the initial implementation phase are still being upheld, or whether they may, in fact, no longer exist. The building blocks synthesised in this study (Table 3) may serve as a starting point for IP teams to reflect on their own IP team meetings within their specific context, and ideally to initiate sustainability-oriented actions. Further research is needed to disentangle the roles of the identified aspects in initial implementation versus sustainability.

### Strengths and limitations

A strength of this study is the IP research team, which consisted of five researchers with primary care experience: one occupational therapist (JS), two physical therapists (DU, EB), one nurse (AM), and one general practitioner (MvB). This facilitated a multi-perspective approach to data collection, analysis, and interpretation, supported by regular reflexive meetings. While the CFS offered conceptually rich domains that supported our data analysis in identifying multiple aspects of sustainability in IP team meetings, it provided limited guidance on how these interrelate and how it fits the specific context of primary health care [13]. At present, no alternative framework appears to offer a better fit for studying sustainability in IP primary care settings [30].

The use of convenience sampling for selecting IP teams may be a limitation, as it could favour the inclusion of teams already open to sustainability efforts, introducing a risk of self-selection bias. At the same time, the included teams were heterogeneous in their characteristics, which may have mitigated this risk. Participation of allied HCPs in the FGDs was limited, which may have reduced transferability to IP teams where allied HCPs are more prominently represented. FGDs proved to be a suitable method, yielding rich and recurring data. Data sufficiency was reached after four FGDs, as no new codes emerged, and the existing categories within the CFS framework were sufficiently covered. Although the moderation of the FGDs helped mitigate potential power-related dynamics, we acknowledge that the FGD-format may still have limited the expression of individual perspectives. As a result, the data may primarily reflect shared experiences and views within IP teams. At the same time, the data also showed that teams were not always aligned in their views, as disagreements were explicitly captured during the discussions. This suggests that, while individual-level perspectives may have been less fully accessible, variation and tension within team perspectives were still represented in the dataset.

## Conclusion

Sustaining appears to be an ongoing process that is closely intertwined with the implementation of IP team meetings and shaped by the dynamic interplay of team-, organisational-, and system-level aspects. Our findings could provide a foundation for developing sustainability strategies based on the building blocks. In particular, there is a need for a mind-shift, a new way of thinking, as professionals often perceive IP team meetings merely as occasions to discuss and coordinate patient care, whereas sustaining also requires attention to teamwork and organisational aspects within the team’s scope of influence. Further research is needed to design and pilot sustainability strategies that are responsive to the resource constraints of IP team meetings in primary care, while remaining robust and flexible enough to adapt to changing circumstances over time.

## Supplementary Material

Supplemental Material

Supplemental Material

## Data Availability

The qualitative data (focus group discussion transcripts) that support the findings of this study contain sensitive information that could compromise participant privacy and are therefore not publicly available. This study is part of a larger project. The project’s metadata describing the datasets are available through DataverseNL (https://doi.org/10.34894/UBOV2G). In line with the FAIR principles, the metadata remain accessible, even though the transcripts themselves cannot be openly shared.

## Data deposition

Data were not deposited in a public repository because they contain identifiable and sensitive participant information.
