# Supplementary material for: A new way of thinking? Exploring professionals’ perspectives on the sustainability of interprofessional team meetings: A focus group study
Source: Eur J Gen Pract. 2026 Aug 10;32(1):2708526. doi: 10.1080/13814788.2026.2708526 (PMC13459306; doi:10.1080/13814788.2026.2708526)
Supplement: Supplementary file 1 [file IGEN_A_2708526_SM8266.docx]

**Supplementary material**

A Questioning route: Focus group discussion

B Unconstrained categorization matrix

**A Questioning route – Focus group discussion (1)**

| **Broad opening question(s)** | **I have just explained the theme of our current focus group:**  **What comes to mind for you?**  **What does it make you think of when you reflect on your own interprofessional team meeting?** | |
| --- | --- | --- |
| **Topics** | **Key-Questions (obligation)** | - **Sub-Questions (optional)** |
| **Facilitators/gains** | **What makes your interprofessional team meeting successful?**  **What keeps your interprofessional team meeting going?** | - *When is the interprofessional team meeting successful for you?* - *What should continue to happen as it does now?* |
| **Barriers (2) to sustainability**  (Aspects of the Consolidated Framework for Sustainability e.g., process-related, financial resources, skills, organizational level, staffing/involvement, etc.) | **What barriers do you experience in relation to your interprofessional team meeting?**  **Which barriers need to be addressed in order to ensure the sustainability of your interprofessional team meeting?** | Organization- / Working structure of interprofessional team meeting:   - How satisfied are you with the structure and organization of your interprofessional team meeting? - How satisfied are you with the way patients are discussed during the meetings? - What kind of care-needs or situations lead to a patient being discussed? - How do you reach decisions as a team regarding patient care (shared decision-making within the team)? - How would you like to evaluate your interprofessional team meeting? - What does the collaboration with, for example, physiotherapists, occupational therapists, speech therapists, and dietitians look like?   Shared goals/values:   - How clear are your shared goals and values? - How could the organization or practice support you in achieving these goals more effectively?   Tasks, roles & responsibilities:   - Which tasks or roles do you consider important for the sustainability of your interprofessional team meeting? Are there any roles or tasks that might be missing now? - Are there things you would like to agree on more clearly to support the sustainability of your interprofessional team meeting? - How well are you informed about the tasks and responsibilities of other members in your team?   Communication and information transfer:   - How well does communication work among you, both inside and outside the interprofessional team meeting?   Patient participation during interprofessional team meetings:   - The goal of the interprofessional team meetings is to coordinate care well for patients with complex needs. How do you involve patients or informal caregivers in this process? |
| **Needs (2) for sustainability**  (Aspects of the Consolidated Framework for Sustainability e.g., process-related, financial resources, skills, organizational level, staffing/involvement, etc.) | **What do you need/consider important for sustaining your interprofessional team meeting?**  **What needs to keep happening as it is now to ensure the long-term sustainability of your interprofessional team meeting?** | - What is currently missing for the sustainability of your interprofessional team meeting? - What must you absolutely keep doing? - Suppose everything were possible (in terms of time, resources, organization, etc.); how would you want to sustain the interprofessional team meeting in the long term? - What could support you in sustaining the interprofessional team meetings between the practice, home care, and allied HCPs? - What would you like to work on to ensure the sustainability of your interprofessional team meetings?   *Moderator note: keep the sub-questions/sub-constructs outlined above in mind!* |

1. Krueger RA. *Focus groups: a practical guide for applied research.* Thousand Oaks (CA): Sage Publications; 2014.
2. Lennox L, Maher L, Reed J. Navigating the sustainability landscape: a systematic review of sustainability approaches in healthcare. *Implement Sci.* 2018;13(1):27.
