# Supplementary material for: A new way of thinking? Exploring professionals’ perspectives on the sustainability of interprofessional team meetings: A focus group study
Source: Eur J Gen Pract. 2026 Aug 10;32(1):2708526. doi: 10.1080/13814788.2026.2708526 (PMC13459306; doi:10.1080/13814788.2026.2708526)
Supplement: Supplementary file 2 [file IGEN_A_2708526_SM8273.docx]

**Supplementary material**

A Questioning route: Focus group discussion

B Unconstrained categorization matrix

**B Unconstrained categorization matrix (1) corresponding to the Consolidated Framework for Sustainability**

| **Sustainability constructs (2)** | | | **Unit-/Meaning of analysis** | | |
| --- | --- | --- | --- | --- | --- |
| **Descriptors** | **Sub-categories** | **Categories*** | **F**  **Facilitators** | **B**  **Barriers** | **N**  **Needs** |
| Involvement of those impacted by the initiative, Engagement; Valuing stakeholder opinions; Influencing the initiative | **1.1 Stakeholder participation** | 1. **The people involved** |  |  |  |
| Leadership depth, distribution, and continuity; Leadership support and engagement; Style; Commitment to and Advocates for the initiative; Experienced and strong leadership | **1.2 Leadership and champions** |  |  |  |  |
| Community links; Networking; Building alliances ; Collaboration with other scientists, organizations, communities etc.; Partnerships | **1.3 Relationships, collaboration and networks** |  |  |  |  |
| Patient centeredness; Patient choice and preferences; Beneficiary perspectives considered; Patient values; Empowerment | **1.4 Patient involvement** |  |  |  |  |
| Sense of ownership; Community/Local ownership; Local control and  management | **1.5 Ownership** |  |  |  |  |
| Power distribution; Professional power; Stakeholder power and priorities; Power exchange | **1.6 Power** |  |  |  |  |
| Meaningful work, role satisfaction, benefits of learning new tasks, increase task significance, job more intrinsically rewarding and personally significant, Enjoyment and fun | **1.7 Satisfaction** |  |  |  |  |
| Broad based participation by the community; Community dialogue; Community voice; Understanding the community, local context, local needs and values; Cultural acceptance; Interest and support | **1.8 Community involvement** |  |  |  |  |
| Building staff capacity to deliver the initiative; Involvement in decision making, planning and development of initiatives; Staff interest; Proportion of staff that contribute; Staff attitudes and acceptance | **1.9 Staff involvement** |  |  |  |  |
| Ability to fit in with organizations, how the intervention ‘conforms’ to existing institutions, integration into routine processes, alignment with strategic goals, administrative policies and procedures | **2.1 Integration with existing**  **programs and policies** | 2. **Organizational setting** |  |  |  |
| Prevailing beliefs and value systems, compatibility of beliefs with initiative, support for improvement, limitations and opportunities created by the organization, workplace climate and cohesion | **2.2 Organizational Values and Culture** |  |  |  |  |
| Resistance from stakeholders to the initiative due to other priorities or competing interests. | **2.3 Opposition** |  |  |  |  |
| Reminders, education boosters, management support, supervision and peer-support, technical assistance; data support | **2.4 Support Available** |  |  |  |  |
| Response to opportunities and challenges, modification, flexibility, monitored and evaluated regularly, adjusted as needed | **2.5 Intervention adaptation and receptivity** |  |  |  |  |
| Viability, Abilities and experience, preparedness, Building capacity/education, Absorptive capacity, Support Service Provision, Ability to learn and adapt to the environment | **2.6 Organizational**  **Readiness and**  **Capacity** |  |  |  |  |
| Staff: Sufficient number of staff; Permanent personnel; Human resources, staff mobility; turnover  Time: Time in schedules, staff time, volunteer time, allocated time  Funding: Money, funding, grants, Donations, Reimbursement | **3.1 General resources (funding, staff, time)** | 3. **Resources** |  |  |  |
| Infrastructure support; Workplace climate and structure, work spaces, materials, supplies and medications; information support systems, software, access to information | **3.2 Infrastructure** |  |  |  |  |
| Environmental considerations; Government policies; Social indicators; Critical events; Technology change; Market forces; Population characteristics; Environmental scans | **4.1 Socioeconomic and political considerations** | 4. **The External Environment** |  |  |  |
| Business cases; Active Communication of main messages and benefits; Publicity- marketing and sales pitch; Newsletters, publications; Success sells; Awareness of the issue | **4.2 Awareness and Raising the profile** |  |  |  |  |
| program diffusion; replication at other sites; spread to other parts of an organization | **4.3 Spread to other organizations** |  |  |  |  |
| Scale of the burden; urgency of the issues; Severity of the issue; relevance to fundamental mandates of the organization; concern for the problem | **4.4 Urgency** |  |  |  |  |
| Tasks, activities, roles taken on, delegation; professional responsibilities; varied roles for participation; allocation of work between individuals; burden to individuals; proportion of staff contributing; clarity of roles and responsibilities; reliance on individuals | **5.1 Accountability of roles and responsibilities** | 5. **Negotiating initiative processes** |  |  |  |
| Consensus, shared goals; clarifying goals, set directions, shared purpose, mutual goal setting, shared expectations | **5.2 Defining Aims and Shared Vision** |  |  |  |  |
| Value of the initiative; legitimacy of the intervention, confidence in the intervention; commitment of stakeholders; staff perceptions of need and quality of innovation; credibility, utility | **5.3 Belief in the initiative** |  |  |  |  |
| Burden (complexity and cost); Program familiarity; simplicity, easy to describe and teach | **5.4 Complexity** |  |  |  |  |
| Positive incentives to participate; individual benefits, perceived effects, higher prestige, increased reputation for an institution, awards, and process performance feedback; rewards; motivation | **5.5 Incentives** |  |  |  |  |
| Work agreement, delegating responsibilities, job descriptions, job roles, skill sets, activities incorporated into day jobs | **5.6 Job Requirements** |  |  |  |  |
| Sufficient number of staff, time to complete tasks in daily schedule, workload is manageable, added effort, reduce waste, division of labour | **5.7 Workload** |  |  |  |  |
|  |  | 6. **Other**   (space for inductive content) |  |  |  |

*One category is missing: ‘The Initiative Design and Delivery’, because it focuses specifically on sustainability constructs of sustainability strategies.

1. Elo S, Kyngäs H. The qualitative content analysis process. *J Adv Nurs.* 2008;62(1):107–15.
2. Lennox L, Maher L, Reed J. Navigating the sustainability landscape: a systematic review of sustainability approaches in healthcare. *Implement Sci.* 2018;13(1):27.
